# From Mitochondrial Myopathy to *SLC18A3*-Related Congenital Presynaptic Myasthenic Syndrome: A Case Report

**DOI:** 10.1159/crn/abyag009

**Published:** 2026-08-28

**Authors:** Anke Geertje Boxum, Dennis Klaas Bos, Gea Drost, Jan-Willem Elting, Tom Jozef de Koning, Sonja Leonie van Ockenburg, Lisette Henriëtta Koens

**Affiliations:** a Department of Neurology, University Medical Center Groningen (UMCG), University of Groningen, Groningen, The Netherlands; b Department of Clinical Neurophysiology, University Medical Center Groningen (UMCG), University of Groningen, Groningen, The Netherlands; c Department of Genetics, University Medical Center Groningen (UMCG), University of Groningen, Groningen, The Netherlands; d Department of Neurosurgery, University Medical Center Groningen (UMCG), University of Groningen, Groningen, The Netherlands; e Pediatrics, Department of Clinical Sciences, Lund University, Lund, Sweden; f Expertise Centre Movement Disorders Groningen, University Medical Centre Groningen, Groningen, The Netherlands; g Department of Endocrinology, University Medical Center Groningen (UMCG), University of Groningen, Groningen, The Netherlands; h Department of Neurology and Clinical Neurophysiology, Martini Hospital, Groningen, The Netherlands

**Keywords:** Congenital myasthenic syndrome, SLC18A3, Repetitive nerve stimulation, Postexercise exhaustion, Case report

## Abstract

**Introduction:**

Recognizing congenital myasthenic syndromes is essential, as appropriate medication can improve muscle weakness.

**Case Presentation:**

We report on a 25-year-old male who was diagnosed with a mitochondrial myopathy based on muscle biopsy in childhood, although genetic testing of the mitochondrial DNA did not show abnormalities. New analysis, including a gene panel, revealed two novel variants in the SLC18A3 gene, and characteristic electromyography findings changed his diagnosis to a congenital myasthenic syndrome. Treatment was started, which significantly improved his quality of life.

**Conclusion:**

Our report underscores the importance of re-evaluating diagnoses in patients without genetic confirmation, so that treatable conditions are not overlooked.

## Introduction

Congenital myasthenic syndromes are a group of rare genetic disorders that impair neuromuscular transmission. They are predominantly inherited in an autosomal recessive manner, although autosomal dominant forms have also been described. They are primarily characterized by limb, ocular and bulbar muscle weakness, along with rapid fatigue following physical activity. However, these symptoms are not unique to congenital myasthenic syndromes and are also observed, for example, in myopathy in mitochondrial disorders. A significant improvement after rest may serve as a crucial indicator of a myasthenic syndrome [1]. Recognizing congenital myasthenic syndromes is essential, as muscle weakness can improve with appropriate medication. We present the case of a 25-year-old male, whose initial diagnosis of mitochondrial myopathy was revised to congenital presynaptic myasthenic syndrome. This revision was based on characteristic clinical neurophysiological findings and identification of a variant in the SLC18A3 gene that has not been reported before. This case report was prepared in accordance with the CARE guidelines. The completed CARE Checklist is provided as online supplementary Material (for all online suppl. material, see https://doi.org/10.1159/crn/abyag009).

## Case Presentation

The patient was born at term, but experienced respiratory insufficiency shortly after birth. He exhibited feeding and swallowing difficulties with choking, barely cried, and suffered from hypotonia, ptosis and external ophthalmoplegia. There was no family history of neurological diseases, and he had a healthy older brother. A congenital myasthenic syndrome was considered, but a neostigmine test was negative. Also, acetylcholine receptor antibodies in the context of neonatal myasthenia gravis came back negative. Repetitive nerve stimulation around the age of 1 year did not show a decrement; however, needle electromyography (EMG) indicated abnormalities consistent with myopathy at that time. Serum lactate was elevated, but other laboratory studies, including CK, were normal. A muscle biopsy revealed low ATP production with complex II–III deficiency, consistent with mitochondrial myopathy. Genetic analysis of mitochondrial DNA was normal. Targeted genetic analysis of nuclear DNA showed a variant c.113_130del,p.(Arg38+ser43del) in *OPA1*, of which the clinical implications were unclear. At the time, whole-exome sequencing (WES) with the use of broad gene panels was not widely available.

In the following years, the patient experienced exercise intolerance, respiratory distress during intercurrent infections, strabismus, and ptosis, for which he underwent multiple surgeries. At the age of 24 years, he visited our clinic due to a tremor, thought to be an enhanced physiological tremor. He told us that after walking 100 meters, he needed to rest for 5–10 min before being able to continue again. He had difficulties chewing and swallowing. Furthermore, there was some intellectual disability. There were no seizures or dysautonomia. Neurologic examination showed bilateral ptosis, more severe on the right side, with strabismus, ophthalmoparesis and saccadic intrusions. Muscle power grading on the MRC scale was 4 in the deltoid and quadriceps muscles; in other muscles, there was no weakness. However, he showed clear fatiguability during the examination. The patient had very low deep tendon reflexes. Besides an enhanced physiological tremor, there were no other movement disorders.

Given the combination of symptoms without a confirmed genetic diagnosis, we performed a WES panel for “muscle disorders”. Two heterozygous variants of uncertain significance (VUS): c.392A>G,p.(Lys131Arg) and c.1089del,p.(Leu354Trpfs*3) in *SLC18A3* (NM_003055.3) were found, suggesting a *SLC18A3-*related congenital presynaptic myasthenic syndrome [1]. Segregation analysis revealed that both parents were heterozygous for one of the two variants.

Subsequently, nerve conduction studies were performed. These showed no significant decrement in the abductor pollicis brevis with slow repetitive stimulation, but did show decrement after exercise up to 90% (Fig. 1). Repeated EMG examination (after the start of treatment) demonstrated decrements in the median and ulnar nerves of 45% and 21%, respectively. Single-fiber EMG demonstrated jitter with a mean consecutive difference of 43 µs initially, increasing to 188 µs, with blockings during the examination (Fig. 2). Using the electrophysiology data, the VUS in our patient were reclassified as likely pathogenic (c.392A>G,p.Lys131Arg) and pathogenic (c.1089del,p.(Leu364Trpfs*3)) in *SLC18A3* (NM_003055.3). Our patient was started on pyridostigmine and noticed increased energy, improved eating and drinking, and enhanced speech.

**Fig. 1.**
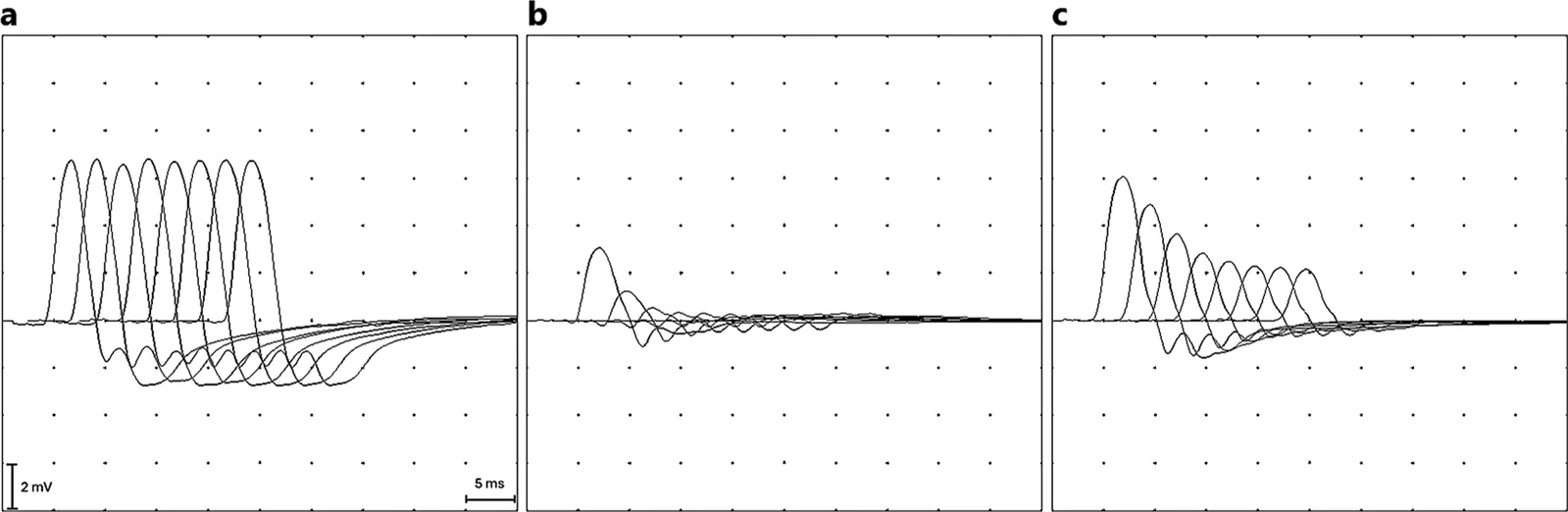
3 Hz repetitive nerve stimulation of the median nerve, with the active electrode placed on the abductor pollicis brevis. Repetitive nerve stimulation after resting (**a**), immediately after 20 s of exercise (**b**), and 3 min later (**c**) is shown. There is no decrement at rest, but after exercise, the decline of the compound muscle action potential (CMAP) becomes apparent, with decrements of more than 90% and 55%, respectively.

**Fig. 2.**
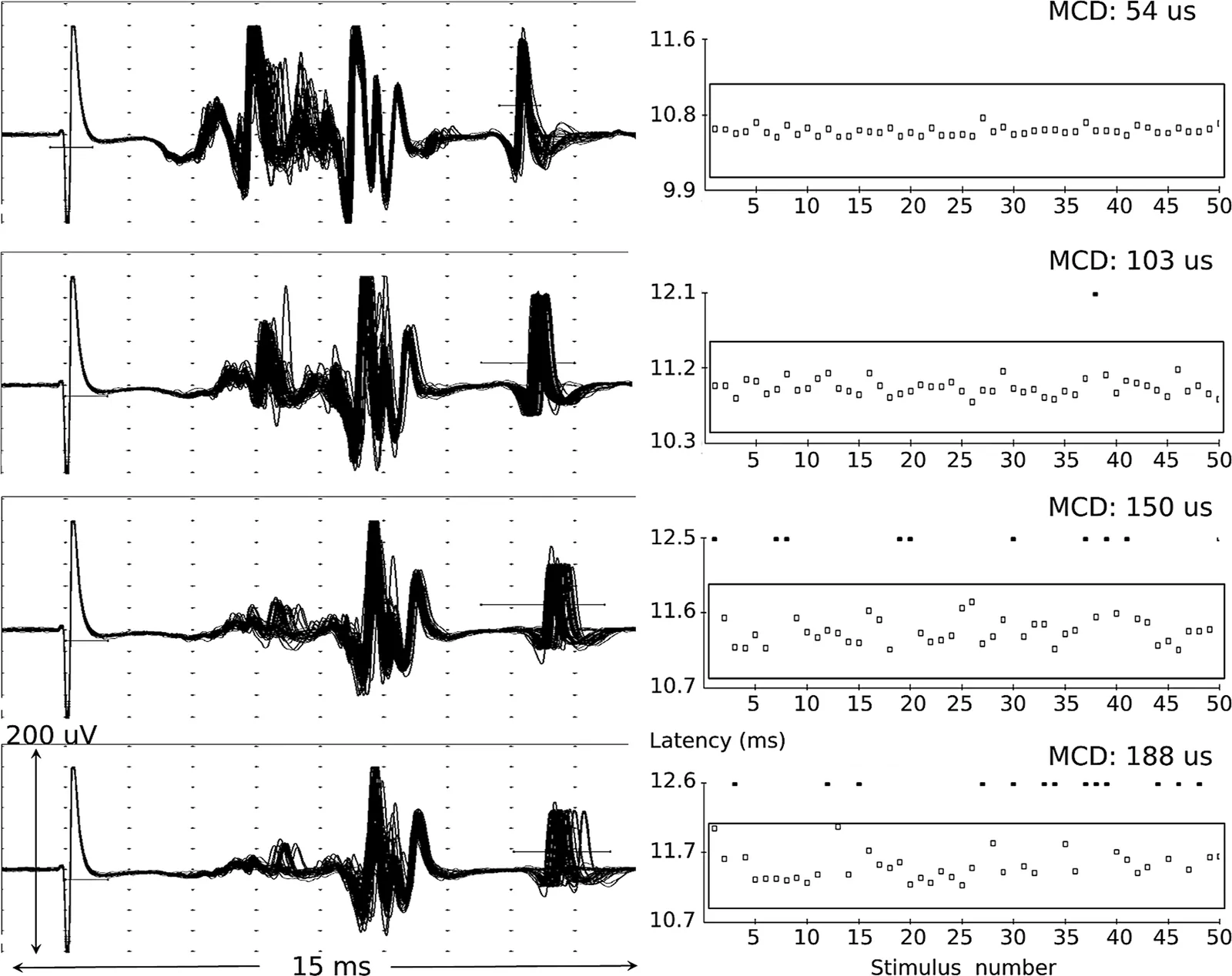
Example of SF-EMG results: the facial nerve was stimulated on the right side at 10 Hz for 5 s. The orbicularis oculi was recorded with a concentric needle. Four successive recordings of the same fibers are shown. Note the progressive increase in jitter and blockings with each new recording, indicating worsening neuromuscular transmission. The right column shows the results of the muscle fiber on the right, in terms of latency vs. stimulus number. Note the increase in mean consecutive difference (MCD). Blockings are depicted as dots at the maximum Y-value in each latency vs. stimulus number graph. SF-EMG, single-fiber EMG.

## Discussion

The SLC18A3 gene, located on chromosome 10q11.23, encodes the vesicular acetylcholine transporter, a protein that regulates the active transport of acetylcholine at the neuromuscular junction [1]. Our patient showed two novel heterozygous VUS in *SLC18A3*. We used segregation analysis, showing that both parents were heterozygous for one of the two variants. Since biallelic mutations in *SLC18A3* are associated with congenital presynaptic myasthenic syndromes [1], these variants could account for the patient’s phenotype.

We used nerve conduction studies to test our hypothesis, which showed a decrement with slow repetitive stimulation after exercise. This is typical for presynaptic disorders and was interpreted as postexercise exhaustion (Fig. 1) [2]. In postsynaptic neuromuscular junction disorders, such as myasthenia gravis, a decrement is usually observed with slow repetitive stimulation alone.

The findings during repetitive stimulation align with those in the few other patients with a defect in *SLC18A3* described in the literature [1, 3–5]. The results of increasing mean consecutive difference and blockings during the single fiber examination (Fig. 2) also underscore postexercise exhaustion and are an indication of disturbed neuromuscular transmission [3, 4]. The results of our neurophysiological tests were convincing, which enabled us to reclassify the VUS in our patient as pathogenic and likely pathogenic.

To conclude, this case report illustrates a patient initially diagnosed with a mitochondrial disorder based on abnormal biochemistry in a muscle biopsy and a variant of unknown significance in a gene related to mitochondrial disease, later identified as having a presynaptic congenital myasthenic syndrome. WES was not widely available at the time of birth. Nowadays, with WES and whole-genome sequencing, we are able to diagnose these patients faster and at a much younger age. It underscores the need for caution when interpreting historical muscle biopsy results without genetic confirmation. Deficiencies in complex II–III are indicative, but not specific for mitochondriopathy. Muscle biopsies typically do not show specific findings in congenital myasthenic syndromes; most cases exhibit no abnormalities, though myopathic findings and defects seen in primary mitochondrial oxidative phosphorylation have been described [6]. Abnormalities are sensitive to training and disuse, which could possibly explain the biopsy results. This case highlights the importance of re-evaluating diagnoses and performing repeated genetic testing to identify treatable disorders, thereby improving quality of life. It also shows that neurophysiological data can help in classifying VUS.

## Statement of Ethics

This retrospective review of patient data did not require ethical approval in accordance with local guidelines. Written informed consent was obtained from the patient for publication of the details of his medical case and any accompanying images.

## Conflict of Interest Statement

The authors have no conflicts of interest to declare.

## Funding Sources

This study was not supported by any sponsor or funder.

## Author Contributions

A.G.B., J.W.E., and L.H.K. initiated the case report. L.H.K., T.J.K., S.L.O., and D.K.B. saw the patient in the outpatient department of metabolic diseases. D.K.B. was involved in genetic analyses. A.G.B., J.W.E., and G.D. performed the electromyography analyses. A.G.B. and L.H.K. drafted the report. All other authors commented on drafts. The final version was approved by all authors.

## Data Availability

The data that support the findings of this study are not publicly available due to privacy reasons but are available from the corresponding author upon reasonable request.
